# Optimization of Serial Modular Continuous Mixing Process Parameters for Natural Rubber Composites Reinforced by Silica/Carbon Black

**DOI:** 10.3390/polym12020416

**Published:** 2020-02-11

**Authors:** Lin Zhu, Xiaolong Tian, Yiren Pan, Tianhao Chang, Kongshuo Wang, Guangzhi Niu, Luqi Zhang, Chuansheng Wang, Wenwen Han

**Affiliations:** 1 College of Electromechanical Engineering, Qingdao University of Science and Technology, Qingdao 266061, China; qustzhulin@163.com (L.Z.); 15165268516@163.com (X.T.); pyr90hot@163.com (Y.P.); chang_tianhao@163.com (T.C.); kongshuo726@163.com (K.W.); ngz15763903965@163.com (G.N.); ezhangluqi@163.com (L.Z.); 2 Shandong Provincial Key Laboratory of Polymer Material Advanced Manufacturing Technology, Qingdao University of Science and Technology, Qingdao 266061, China; 3 National Engineering Laboratory for Advanced Tire Equipment and Key Materials, Qingdao University of Science and Technology, Qingdao 266061, China; 4 Academic Division of Engineering, Qingdao University of Science and Technology, Qingdao 266061, China

**Keywords:** serial modular continuous mixing, process parameters, optimization, silica/carbon black, natural rubber

## Abstract

In the tire industry, the combination of carbon black and silica is commonly utilized to improve the comprehensive performance of natural rubber so as to realize the best performance and cost-effectiveness. The corresponding mixing is divided into three processes (initial mixing, delivery, reactive mixing) by the serial modular continuous mixing method, thus achieving more accurate control of the mixing process, higher production efficiency and better performance. Moreover, the optimization of serial modular continuous mixing process parameters can not only improve the performance of composite materials, but help people understand the physical and chemical changes and the reinforcing mechanism of fillers in the mixing process. In this paper, the relationship among the parameters of eight processes and filler network structure, tensile strength, chemical reinforcing effect and tear resistance was explored through experiments. The deep causes of performance changes caused by parameters were analyzed. Consequently, the best process condition and the ranking of the influencing factors for a certain performance was obtained. Furthermore, the best preparation process of natural rubber (NR)/carbon black/silica composite was achieved through comprehensive analysis.

## 1. Introduction

Natural rubber, a material that is superior to synthetic rubber in terms of elasticity and mechanical properties, is usually utilized in high-quality tires, conveyor belts, high-quality rubber products, etc. [1,2,3]. As green tires spring up around the world, silica is widely utilized in tire treads in order to improve the tear resistance, enhance the wet skid resistance of tires and reduce rolling resistance [4,5,6,7,8]. However, the application of silica alone as a reinforcing filler results in an increase in production costs, as well as the decline of tire wear resistance and antistatic property. Therefore, the combination of carbon black/silica in the tire industry is generally used to achieve the best performance and cost effectiveness [9,10,11,12].

In modern industry, batch internal mixers are usually utilized to prepare carbon black/silica/rubber composite materials. After the first stage of mixing and dumping, the second stage of mixing is needed in order to realize a sufficient silanization reaction and control the heat load of rubber mixing. However, achieving acceptable performance also wastes energy and time [13,14,15,16,17,18,19]. In recent years, many scholars and companies have contributed to developing continuous mixers, such as the Farrell continuous mixer, the twin-shaft continuous mixer, the Buss Kneader continuous mixer, and the co-rotating twin screw continuous mixer [20,21,22,23]. Nevertheless, due to the structural limitations of the feeding port of the above continuous mixers, only powdery and granular materials are able to be added, except for block rubber. As a result, these continuous mixers are mostly applied in the plastic industry [24,25,26,27,28]. In addition, even though granular rubber is used as the raw material regardless of cost, the accuracy of the ratio of raw rubber and filler is hard to ensure with these continuous mixing methods. Consequently, based on the requirements of modern industrial continuous rubber mixing, a serial modular continuous mixer (SMCM) has been designed by Professor Wang [29]. The modular design ensures the accuracy of the ratio, and also has a good residence time, temperature control, and exhaust. The core components are the initial mixing rotors and the core reaction mixing twin rotors. The special geometry causes these components to produce elongational forces except shear stress to the compound, which enhances the dispersion effect and reaction extent. Previous studies have shown that, compared to two-stage mixing, the serial process comprehensively improves the dispersion, mechanical properties, and dynamic mechanical properties of silica/rubber vulcanizate [30].

Nowadays, many studies on the mixing processes of reinforcing natural rubber composite materials with silica/carbon black are all based on traditional internal mixers or open mill [31,32,33,34,35,36,37,38,39]. For SMCM, mixing is divided into three closely related processes: initial mixing, delivery and reactive mixing. Specifically, the initial mixing module aims to realize the rapid incorporation, dispersion and distribution of the filler in the matrix rubber under the premise of ensuring the accurate mixing ratio. Only when the initial mixing rubber reaches proper mixing effect, can the downstream reaction, which is the module where the constant temperature silanization reaction takes place, proceed smoothly. In addition, reasonable control of reaction time and temperature is the key to improve the performance of composite materials. The modular structure increases the uncertainty of the mixing process, although it increases the process parameter variables that facilitate more precise control of the mixing. Therefore, it is significant to research the parameters optimization of the series continuous mixing. In this paper, the effect of the parameters of eight processes on the filler network structure, tensile strength, chemical reinforcing effect and tear resistance of natural rubber (NR)/carbon black/silica composite was explored through orthogonal experiment. Consequently, the best process condition and the ranking of the influencing factors for a certain performance was obtained. Furthermore, the best preparation process of NR/carbon black/silica composite was achieved through comprehensive analysis.

## 2. Materials and Methods

### 2.1. Formula and Materials

Rubber formulations and suppliers are shown in Table 1.

### 2.2. Equipment

**Figure 1:**
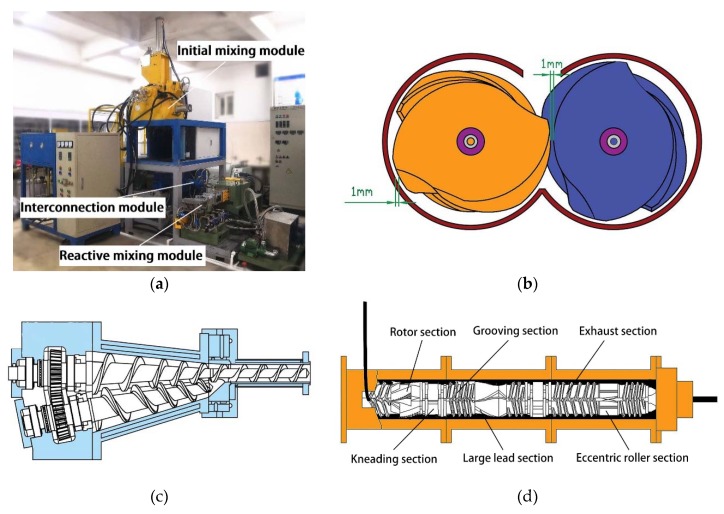
Serial Modular Continuous Mixer (**a**) Photo of Serial Modular Continuous Mixer; (**b**) Schematic diagram of the initial mixing rotors; (**c**) Schematic diagram of interconnection module; (**d**) Schematic diagram of the reactive mixing module.

The SMCM, which is a novel piece of continuous mixing equipment, was invented by Wang for rubber processing [29]. A picture of the SMCM is shown in Figure 1a. It is mainly composed of an initial mixing module, an interconnection module, and a reactive mixing module.

**Figure 2:**
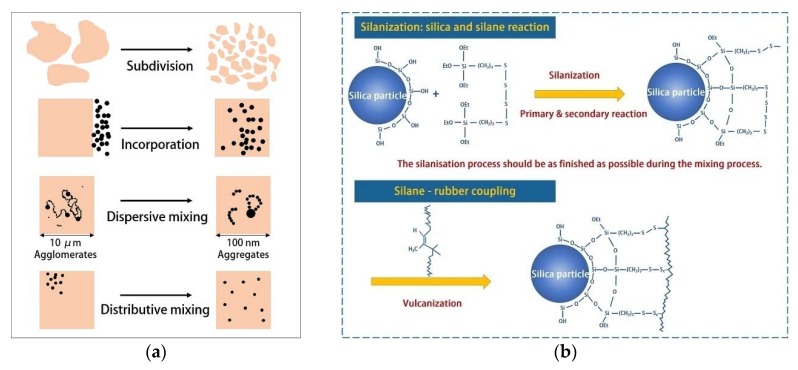
Primary responsibilities of the initial mixing module and the reactive mixing module. (**a**) Initial mixing module: subdivision, incorporation dispersive mixing and distributive mixing; (**b**) Reactive mixing module: silanization reaction.

The batch feeding method adopted in the initial mixing module can maximize the accuracy of the ratio of raw rubber and filler. In the initial mixing module, the rubber subdivision, filler incorporation, dispersive mixing and distributive mixing are major processes, as shown in Figure 2a [40,41]. Additionally, Figure 1b shows a new Co-flow rotor configured in the initial mixing module. Specifically, the shape of new Co-flow rotor is similar to a traditional tangential rotor, which can generate a strong shear effect on the rubber and promote dispersion mixing. However, the installation center distance is small, showing as intermeshing type. Additionally, the edge of the rotor meshes with the base circle of the other rotor (the gap is only 1 mm), producing a squeeze, tear and axial mixing effect on the rubber similar to the intermeshing rotor and greatly improving the distribution mixing effect. Therefore, the new Co-flow rotor is suitable for the dispersion mixing and distribution mixing of rubbers.

The Figure 1c is a structural diagram of the interconnection module, which connects the upstream initial mixing module and the downstream reactive mixing module and contains the function of buffering rubber and continuous feeding. After the upstream initial mixing is completed, the rubber is continuously fed into the reactive mixing module through the transfer module.

The reactive mixing module is mainly responsible for the silanization reaction shown in Figure 2b. As shown in Figure 1d, three independent temperature control systems that can be set to different temperatures as required is included in the barrel of the reaction module. The large effective contact area between the barrel and the material ensures heat transfer efficiency and consequently offers effective control over reaction temperature. In addition, a series of functional elements are installed on the twin rotor to improve the dispersion and distribution effects of the filler. These components mainly include: (1) four-wing serrated rotors and a combined kneading block mainly applied to promote the dispersion effect; (2) large lead rotors and grooving threads to improve the distribution effect; (3) an eccentric roller designed based on the principle that the low viscosity materials are dispersed well by the tension effect at the later stage of mixing. Consequently, these functional elements boost the dispersion and distribution of the filler, thus promoting the reinforcing effect of carbon black and maintaining the continuous reaction by increasing the contact area of silica and the coupling agent.

In addition to SMCM, the following devices were also used in the experiment: Mill, XK-160E (Dalian Rubber and Plastics Machinery Co., Ltd., Dalian, China); Flat-Panel Vulcanizer, QLB-400X400X2 (Qingdao Yadong Machinery Group Co., Ltd., Qingdao, China); Rubber Process Analyzer, RPA2000 (ALPHA Technologies, Hudson, OH, USA); Universal Tester, AI-7000-MGD (GOTECH Testing Machines Inc., Taichung, China).

### 2.3. Sample Preparation

In this paper, an orthogonal experiment with four horizontal and nine factors, namely L32 (4^9^), was set up to explore the optimal process parameters for preparing NR/carbon black/silica composite by serial modular continuous mixing. Specific factors and corresponding levels are shown in Table 2. Columns A–H represent specific factors. The last column is a blank term, which has no specific factors and is used to reflect the errors caused by random factors. It is denoted as the Error term. The detailed experimental arrangement is shown in Table 3. As can be seen from the table, when factor I is at the level of (1), the four levels of factors A–H appear uniformly. $\bar{K_{1}}$-$\bar{K_{4}}$ represents the average of indicators at each level of each factor. $\bar{K_{1}}$ of factor I represents an average of all factors at various levels. $\bar{K_{2}}$,$\bar{K_{3}}$,$\bar{K_{4}}$ are the same as $\bar{K_{1}}$. Due to the existence of random error, $\bar{K_{1}}$, $\bar{K_{2}}$,$\bar{K_{3}}$ and $\bar{K_{4}}$ of factor I are different, so the range I is used to represent the random error value. When range (factors) > range (I), it can be considered that this factor has an impact on the result. The larger the range, the more significant the effect on the result.

All samples are prepared according to the following process:

(1) Add rubber and mix for 15 s; (2) Add silica and the rest additives, mix for 15 s; (3) Add carbon black, and mix for 15 s; (4) Sweep; (5) Mix to the end of initial mixing time and discharge; (6) Feed rubber into the reactive mixing module through the interconnection module; (7) Silanization reaction occurs in the reaction module and extrude continuously at the die; (8) Leave for 4 h and added vulcanization on an open mill; (9) Leave for 8 h and then vulcanize at the condition of 150 °C/10 MPa × T90 (min).

## 3. Results and Discussion

In this paper, 32 samples were tested for Payne effect (Δ*G*’), tensile strength, reinforcement index M300/M100, and tear strength. Specifically, the rubber processing analyzer was adopted to scan the sample for the test of Payne effect, in which the scanning conditions were set at 60 °C, the scanning frequency was 1 Hz, and the scanning range was 0.28%–40%. Three groups were tested for each sample, and the average was taken as the final test result. A universal testing machine was adopted to carry out tensile and tear tests on 32 groups of samples based on the requirements of GB/T528-2009 and GB/T 529-2008. Tensile and tear tests were performed after vulcanization for 20 h. The gripper of the universal machine moves at a speed of 500 ± 50 mm/min. The tensile specimen is dumbbell type (type 1, 25 mm), and each sample was used to test five groups. If the specimen broke outside the narrow part, the experimental results were discarded and another specimen was taken for repeated experiments. The average of the five groups was taken as the tensile test result for the sample. The tear test was conducted with an unnicked angle tear specimen. Five groups were tested with each sample and the average was taken as the final test result.

### 3.1. Effect of Process Parameters on Filler Network

Generally, the interaction between fillers is characterized by the Payne effect, that is, a phenomenon in which the storage modulus gradually decreases with the increase of strain at a constant temperature and frequency [42]. Therefore, the Payne effect is usually quantified with $\Delta G^{\prime }$. In this experiment, it was calculated according to the following formula:

$$\Delta G^{\prime }=G^{\prime }\left(0.28\%\right)-G^{\prime }\left(40\%\right)$$

**Figure 3:**
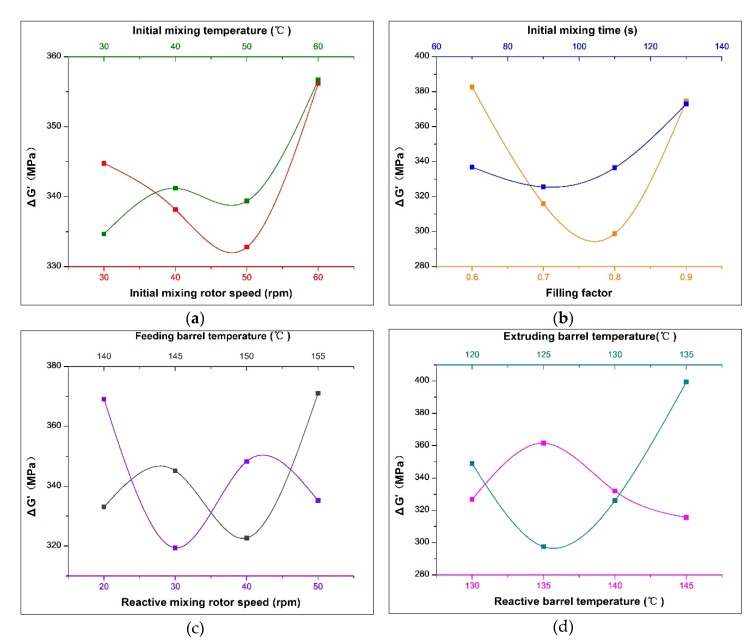
Change trend of the Payne effect with various factors: (**a**) Effect of initial mixing rotor speed and initial mixing temperature on Payne effect; (**b**) Effect of filling factor and initial mixing time on Payne effect; (**c**) Effect of reactive rotor speed and feeding barrel temperature on Payne effect; (**d**) Effect of reactive barrel temperature and extruding barrel temperature on Payne effect.

Notably, the smaller the Payne effect, the less the network structure between fillers, indicating that fillers are better dispersed in the rubber. The range analysis of the orthogonal experiments based on the Payne effect test results is shown in Table 4, and the change trend of the Payne effect with various factors is shown in Figure 3.

**Figure 4:**
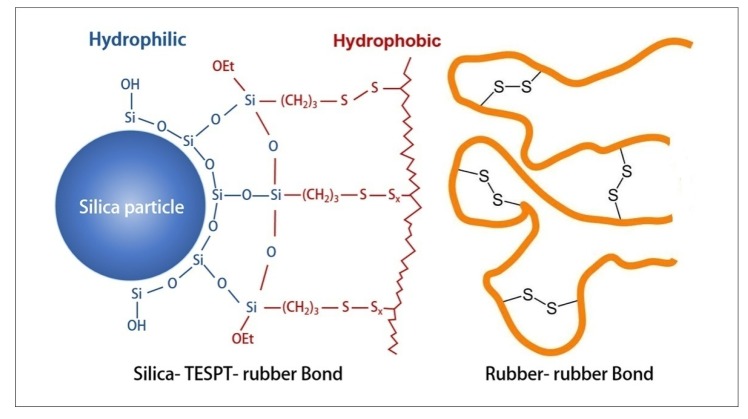
Silane–rubber bond and rubber–rubber bond.

As shown in Table 4, based on the comparison between range (factor) and range (error), it can be concluded that other factors have a great influence on Payne effect, except for the initial mixing rotor speed, the initial mixing temperature and the reactive barrel temperature. The barrel temperature of the extruding zone has the greatest influence on the Payne effect. Specifically, Figure 3d shows that the Payne effect decreases first and then increases with the barrel temperature of the extruding zone gradually increases from 120 °C. When the barrel temperature of the extruding zone reaches about 126 °C, the force between fillers is the smallest and the fillers are best dispersed. This is because the temperature of the rubber compound has reached above 145 °C before passing through the extruding zone. Additionally, the rubber compound reaches a continuous exothermic state due to the previous mechanochemical action. Therefore, the barrel of the extruding zone creates a cooling effect on the rubber compound. However, when the temperature reduction of the barrel is severe, the temperature of the rubber compound decreases rapidly, the silanization reaction speed slows down, and the silica without coupling effect agglomerates in large quantities under the action of polarity. As the temperature of the rubber increases, both of the silanization reaction rate and the hydrophobicity degree of the silica improves and the interaction between rubber and fillers increases, resulting in a decrease in the Payne effect. However, if the barrel temperature in the extruding zone is too high and consequently causes the rubber temperature to reach above 155 °C, the S element contained in TESPT will prematurely crosslink with rubber, forming the silane–rubber bond and the rubber–rubber bond shown in Figure 4. As a result, the Mooney viscosity of the rubber is increased by these bonds, and the movement of the rubber molecular chain is obstructed, resulting in a bad influence on the dispersion of fillers and enhance the Payne effect. Therefore, the proper barrel temperature of the extruding zone is the key to reduce the interaction between fillers.

According to the range analysis, the filling factor of the initial mixing module is the second major factor that affects the Payne effect. As shown in Figure 3b, when the value of the filling factor is small, because the rubber compound keeps in a state of discontinuous flow in the internal mixer, only a small amount of it is forced to pass through the gap between the rotor edge and the inner wall of the internal mixer. As a result, the rubber compound is subjected to less effective shear force, resulting in a higher Payne effect. With the increase of the filling factor, the shear action on the rubber compound enhances and the network between the filler breaks. However, when the filling factor is too high, it is difficult for the fillers to bite and accumulate at the feeding port of the internal mixer. Additionally, since the rotor has a limited turning and folding effect on the rubber compound, the fillers are hard disperse and mix, resulting in undesired filler dispersion effects. As shown in the experimental results, the Payne effect is found to be the lowest when the filling factor is around 0.77.

In addition to the above two factors, the Payne effect is affected by the initial mixing time, the speed of the reaction rotor, and the temperature of the feeding barrel, which basically have the same importance. As shown in Figure 3b, with the extension of the initial mixing time, the Payne effect first decreases and then increases, reaching its minimum value at around 95 s. Firstly, with the extension of mixing time, the wettability and dispersion of the filler are promoted by the high temperature softening and the input of mechanical energy of the rubber, leading to the reduction of the Payne effect. Nevertheless, the continued extension of mixing time increases the network structure of the filler. The reasons for this can be divided into two aspects: one is that the increase in material fluidity leads to a reduction in the dispersion of mechanical forces, the other is that the initial mixing temperature is far from the starting temperature of the silanization reaction, so the surface of the silica has not been modified. In addition, the high temperature exacerbates the polar agglomeration of silica, resulting in an increase in the filler network. This situation gradually improves with continued mechanical dispersion and silanization.

The reaction time and the strength of the mechanical force are determined by the speed of the reaction rotor. As shown in Figure 3c, the optimal speed of the reaction rotor is 30 rpm. When the speed is too low, the dispersion force of the dual rotor is low, the contact area between carbon black and TESPT is small, and the degree of modification of silica cannot be promoted with a long reaction time. With the increase of the rotation speed, the dispersion and reaction reach the optimal balance.

As shown in Figure 3c, the filler network structure reaches its lowest point at a feeding barrel temperature of 150 °C. When the barrel temperature in the feeding zone is too low, the silanization reaction of the rubber cannot start immediately, and the total reaction time decreases, resulting in a low degree of silanization of silica, agglomeration of unmodified silica, and a high Payne effect. As the temperature gradually increases, the reaction degree increases, while the filler aggregation decreases. When the barrel temperature in the feeding zone is too high, scorching of the rubber occurs too early due to the S contained in TESPT, thus hindering the dispersion and reaction of the filler and enhancing the Payne effect again.

Other process parameters have little effect on Payne effect. In general, according to the analysis of the test results of the Payne effect, the best process parameters for preparing NR/carbon black/silica composite materials by serial modular continuous mixing method are (in descending order of major and minor): extruding barrel temperature 126 °C, filling factor 0.77, reactive mixing rotor speed 30 rpm, feeding barrel temperature 150 °C, initial mixing time 95 s, reactive barrel temperature 145 °C, initial mixing rotor speed 48 rpm, initial mixing temperature 30 °C.

### 3.2. Effect of Process Parameters on Tensile Strength

**Figure 5:**
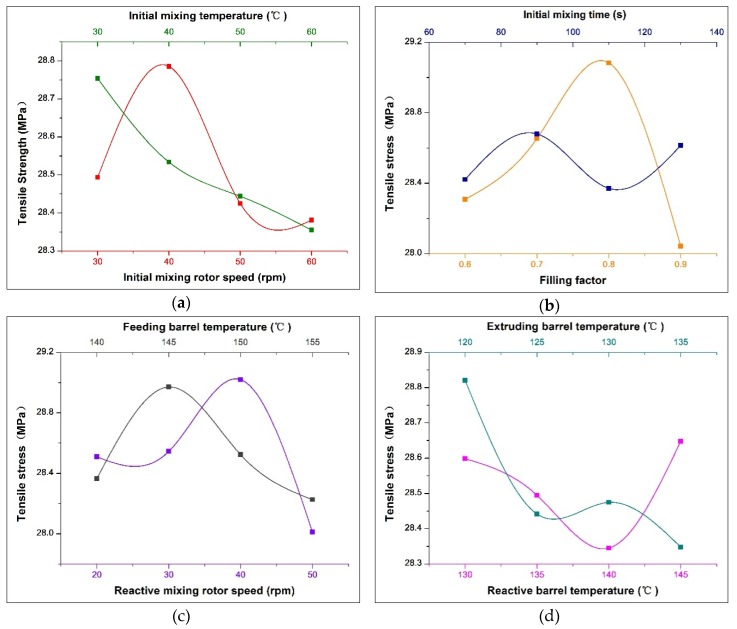
Change trend of the tensile strength with various factors: (**a**) Effect of initial mixing rotor speed and initial mixing temperature on tensile strength; (**b**) Effect of filling factor and initial mixing time on tensile strength; (**c**) Effect of reactive rotor speed and feeding barrel temperature on tensile strength; (**d**) Effect of reactive barrel temperature and extruding barrel temperature on tensile strength.

The tensile strength is one of the important indicators to measure the quality of rubber. The range analysis of orthogonal experiments based on the tensile strength test results is shown in Table 5, and the change trend of the tensile strength with various factors is shown in Figure 5.

According to the range analysis, the filling factor, reactive mixing rotor speed and feeding barrel temperature have significant influence on tensile strength, of which the filling factor is the most important. As shown in Figure 5b, as the filling factor increases, the tensile strength first increases and then decreases, with a peak at 0.78. The reason is that the mechanical force of the rotor on the rubber material is small at low filling factors, which causes poor dispersion results and poor filler reinforcement effect. On the other hand, an excessively high filling amount will increase the difficulty of the biting and dispersion of the filler and reduce the reinforcing effect. Therefore, a suitable filling factor is needed to ensure the good dispersion and reinforcing effect of the filler. 

**Figure 6:**
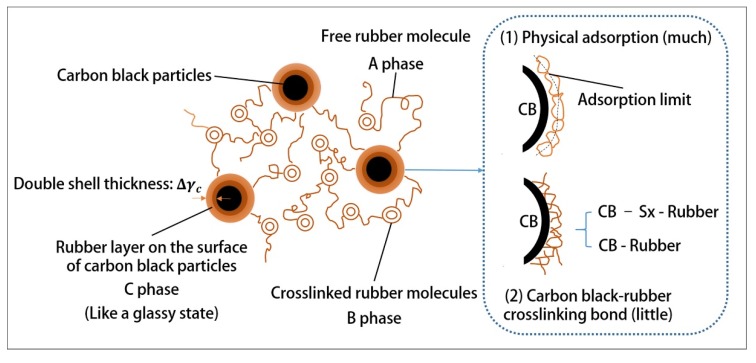
Reinforcing mechanism of carbon black.

The speed of the reaction rotor is the second most important factor that affects the tensile strength. As shown in Figure 5c, the tensile strength is the biggest when the speed of the reaction rotor reaches 40 rpm. In this formula, there are two supplements, carbon black and silica, which hold different reinforcement mechanism. Specifically, as shown in Figure 6, the rubber filled with carbon black contains three phases, ABC, of which the C phase plays the role of a skeleton, acting as the key to reinforcement. Most of the C phases are formed by the physical adsorption of carbon black, while a small part is formed by chemical bonding [43]. However, the reinforcing effect of silica is achieved by chemical reaction in Figure 2b, forming a chemical bonding of silica–silane–rubber. Therefore, the reinforcing effect of the filler in the formula is influenced by interaction of the dispersion of the filler and the degree of silanization reaction. On the one hand, the shear force is affected by the reaction speed, showing that the faster the speed, the greater the mechanical dispersion force. On the other hand, the silanization reaction of silica is affected by the reaction speed, showing that the faster the speed, the shorter the reaction time. As a result, an equilibrium point is necessary to achieve the optimal reinforcing effect. As shown in the experimental results, the optimal rotor speed is 40 rpm. Notably, in comparison to Figure 3c, when the speed of the reaction rotor reaches 30 rpm, the lowest Payne effect is achieved, but the optimal reinforcing effect cannot be produced. It can be inferred that due to the high breakage degree of the rubber molecular chain at 40 rpm, more reactive free radicals are generated, resulting in the formation of the C phase through combination with the active point on the surface of carbon black, resulting in higher tensile strength.

The feeding barrel temperature is the third factor that has an important effect on tensile strength. As shown in Figure 5c, with increasing feeding barrel temperature, the tensile strength first increases and then decreases, and reaching a peak at about 145 °C. This is because the degree of silanization reaction and the chain breakage degree at high temperature of the rubber are affected by the feeding barrel temperature, which then affects the tensile strength. Specifically, excessive low temperature is not conducive to the silanization reaction, resulting in the reduction of the silica reinforcing effect. On the other hand, excessive temperature will cause excessive chain breakage of the rubber under high-temperature oxidation, reducing the strength of the rubber.

The degree of effect of the remaining process parameters on the tensile strength is basically the same. According to the analysis of the test results of tensile strength, the best process parameters for preparing NR/carbon black/silica composite materials by serial modular continuous mixing method are (in descending order of major and minor): filling factor 0.78, reactive mixing rotor speed 40 rpm, feeding barrel temperature 145 °C, extruding barrel temperature 120 °C, initial mixing rotor speed 40 rpm, initial mixing temperature 30 °C, initial mixing time 90 s, reactive barrel temperature 145 °C.

### 3.3. Effect of Process Parameters on Chemical Reinforcement

**Figure 7:**
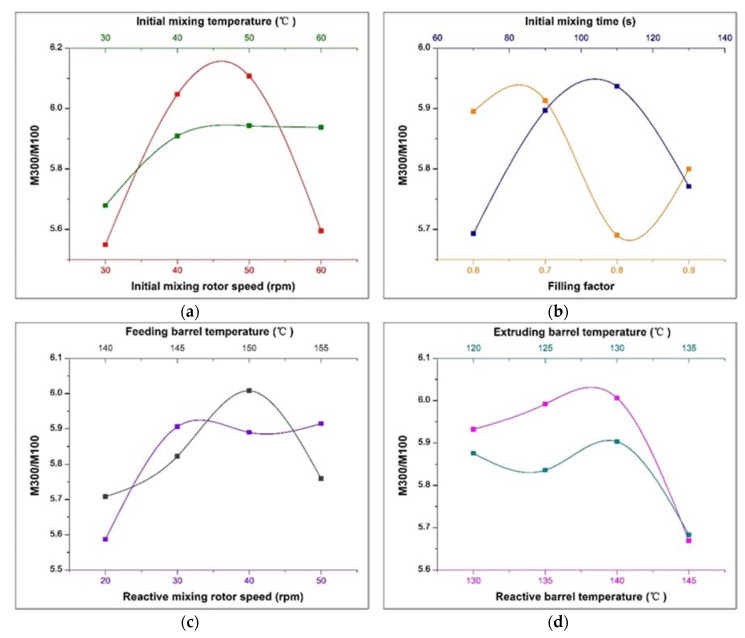
Change trend of the M300/M100 with various factors: (**a**) Effect of initial mixing rotor speed and initial mixing temperature on M300/M100; (**b**) Effect of filling factor and initial mixing time on M300/M100; (**c**) Effect of reactive rotor speed and feeding barrel temperature on M300/M100; (**d**) Effect of reactive barrel temperature and extruding barrel temperature on M300/M100.

The reinforcement index M300/M100 (300% modulus divided by 100% modulus) has a positive correlation with the rubber chemically bonded with vulcanizate. The interfacial area available for chemical bonding of the polymer is the dominant factor for reinforcement [44]. For silica/carbon black/NR composites, the chemical reinforcement mainly comes from silica, because the reinforcement of carbon black mainly depends on the principle of physical adsorption. The optimal silica reinforcement process conditions are obtained by analyzing the effect of process parameters on the reinforcement index M300/M100. The range analysis of orthogonal experiments based on the tensile strength test results is shown in Table 6, and the change trend of the tensile strength with various factors is shown in Figure 7.

According to Table 6, through the comparison between range (factor) and range (error), it can be concluded that all factors have an impact on M300/M00. The initial mixing speed occupies the most important factor that affects M300/M100. The Figure 7a shows that as the initial mixing speed increases, the M300/M100 increases first and then decreases, and the peak value appears at around 45 rpm. In the initial mixing stage of serial continuous mixing, the rubber does not undergo silanization reaction due to the low temperature. The dispersion of the fillers is affected by the speed of the initial mixing, resulting in the variation of the reinforcement index. Specifically, the rubber is subjected to low mechanical force at low speeds, leading to a poor dispersion effect of the fillers. With increasing rotation speed, the mechanical dispersion effect is improved. The silanization reaction occurs quickly after the initial mixing rubber enters the reaction module, resulting in an enhancement of chemical bonding. However, when the rotation speed is excessive high, the dispersion of the filler decreases, resulting in a reduction in reinforcement index. As shown in Figure 3a, the change in the Payne effect also proves that the dispersion of the filler becomes worse when the initial mixing speed is excessively high. This can be attributed to the following two reasons: On the one hand, the Mooney viscosity of the rubber is sharply reduced by the high speed. Although it is beneficial to the infiltration and distribution of the filler, the mechanical dispersion of the filler agglomerates is weakened. On the other hand, the polar agglomeration of the unmodified silica is exacerbated due to the increase of temperature. Therefore, the reinforcing index is reduced by the increase of the filler aggregates network.

The temperature of the reaction barrel, the speed of the reaction rotor, and the temperature of the feeding barrel are also factors that have a great effect on the reinforcement index M300/M100. The three factors are directly related to the silanization reaction and affect the degree of silanization. According to Figure 7d, as the temperature of the reactive barrel increases, the reinforcement index first increases and then rapidly decreases, reaching a maximum at about 140 °C. Similarly, the temperature of the feeding barrel has the same trend, with a peak at about 147 °C, as shown in Figure 7c. This is because whether the temperature of the rubber is suitable for the silanization reaction is determined by these two factors. The proper temperature of the barrel can promote the silanization reaction and improve the reinforcement index. Furthermore, Figure 7c shows that the reinforcement index increases first with the increase of the speed of the reaction rotor, and then remains stable. When the speed is excessive low (below 30 rpm), the mechanical dispersion force is small. The contact area between silica and TESPT is small, and the degree of the silica modification cannot be improved by a long reaction time. With the increase in rotation speed, the degree of dispersion and reaction degree are improved. When the speeds of the reaction rotor are 30/40/50 rpm, respectively, the reinforcement indexes reach the highest and basically the same.

Other process parameters have little effect on reinforcement index M300/M100. In general, according to the analysis of the test results of M300/M100, the best process parameters for preparing NR/carbon black/silica composite materials by serial modular continuous mixing method are (in descending order of major and minor): initial mixing rotor speed 45 rpm, reactive barrel temperature 140 °C, reactive mixing rotor speed 30–50 rpm, feeding barrel temperature 147 °C, initial mixing temperature 40–60 °C, initial mixing time 100 s, filling factor 0.68, extruding barrel temperature 128 °C.

### 3.4. Effect of Process Parameters on Tear Strength

**Figure 8:**
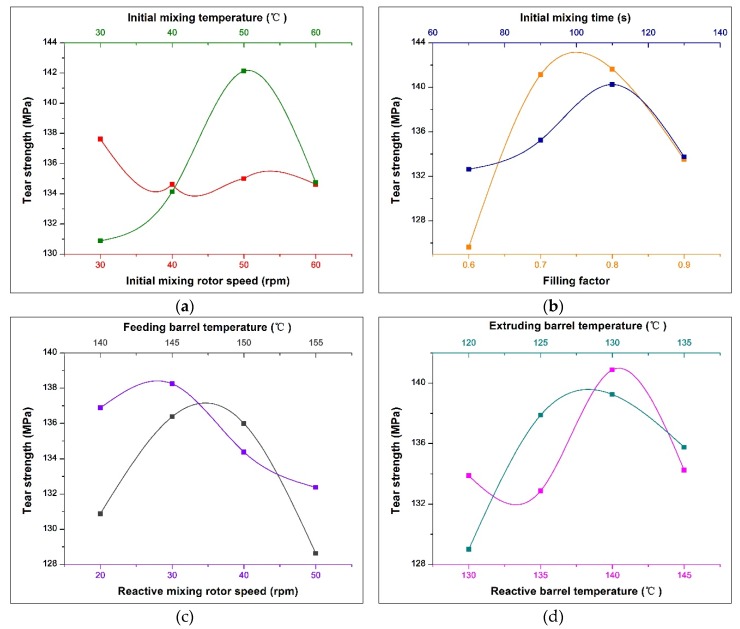
Change trend of the tear strength with various factors: (**a**) Effect of initial mixing rotor speed and initial mixing temperature on tear strength; (**b**) Effect of filling factor and initial mixing time on tear strength; (**c**) Effect of reactive rotor speed and feeding barrel temperature on tear strength; (**d**) Effect of reactive barrel temperature and extruding barrel temperature on tear strength.

The tear resistance of the rubber and the chipping resistance and anti-collapse ability of the tire can be improved by the addition of silica. In this paper, the effect of process parameters on the tear strength is studied. The range analysis of orthogonal experiments based on the tear strength test results is shown in Table 7, and the change trend of the tear strength with various factors is shown in Figure 8.

According to the analysis in Table 7, the range (error) is 2.71, indicating that other factors have a certain influence on tear strength except the initial mixing rotor speed. The filling factor has the greatest influence on the tear strength. When the filling factor is around 0.75, the tear strength reaches its maximum value, which is similar to the trend shown in the Payne effect. It can be inferred that the tear strength is changed by the variation of the filler dispersion. The specific cause analysis is shown in Section 3.1.

The initial mixing temperature is the second most important factor that affects the tear strength. Figure 8a shows that as the initial mixing temperature increases, the tear strength first gradually increases, and then decreases, reaching a peak at 50 °C. In addition to the filler reinforcement, the tear strength is also greatly affected by crosslinking density. According to Figure 3a, when the initial mixing temperature is increased from 30 to 50 °C, the Payne effect increases slightly, which has little effect on the tear strength. However, with the increase of temperature, the S in TESPT will undergo an early vulcanization reaction with rubber, forming a silane–rubber or rubber–rubber bond, which increases the crosslinking density and improve the tear strength. Additionally, when the mixing temperature is raised to 60 °C, the filler aggregates increase significantly, as shown in Figure 3a. Furthermore, the high-temperature oxidation chain breakage of the rubber intensifies, and the degree of tear strength decreases under the combined effect.

The extruding barrel temperature, the reactive barrel temperature and the feeding barrel temperature are the third, fourth, and fifth factors that affect the tear strength. The temperature of the rubber in the reactive mixing module is affected by these three factors. As shown in Figure 8c,d, when the extruding barrel temperature, the reactive barrel temperature, and the feeding barrel temperature reach 128, 140 and 147 °C, respectively, the optimal reinforcement index and tear strength are obtained. At this point, the temperature of the material can be kept between 145 and 155 °C, providing a suitable temperature for silanization. Therefore, the tear strength is greatly affected by the degree of silica silanization.

According to the analysis of the test results of tear strength, the best process parameters for preparing NR/carbon black/silica composite materials by serial modular continuous mixing method are (in descending order from major to minor): filling factor 0.75, initial mixing temperature 50 °C, extruding barrel temperature 128 °C, reactive barrel temperature 140 °C, feeding barrel temperature 147 °C, initial mixing time 110 s, reactive mixing rotor speed 30 rpm, initial mixing rotor speed 30 rpm.

### 3.5. Optimal Preparation Process Paraments of NR/Carbon Black/Silica Composite

Because complex physical and chemical changes occur in rubber composite materials during mixing, the different properties of materials will show different trends when the mixing process parameters are changed. Therefore, it is not possible to achieve optimal values for all the properties at the same time when mixing. In this paper, the four properties of filler–filler interaction, tensile strength, chemical reinforcing effect and tear strength are comprehensively considered, and the optimal process parameters are analyzed. The selection of the optimal process parameters corresponding to each property are summarized in Table 8, and the order of the degree of influence of the parameters on the performance is marked. Based on the principle of giving priority to important effects, the optimal series modular continuous mixing process parameters of reinforcing natural rubber composite materials with silica/carbon black are determined:

Initial mixing rotor speed 45 rpm, initial mixing temperature 50 °C, filling factor 0.77, initial mixing time 95 s, reactive mixing rotor speed 40 rpm, feeding barrel temperature 147 °C, reactive barrel temperature 140 °C, extruding barrel temperature 128 °C.

## 4. Conclusions

The optimization of the process parameters of serial modular continuous mixing is a way to achieve accurate control of the mixing process and help people better understand the whole process of rubber mixing and the mechanism of material property changes. In this paper, the following conclusions were obtained through parameter optimization experiments:

(1) For rubber composite materials, the process conditions for achieving the optimal value for each property are different. When a certain parameter changes, different trends are shown in different properties. In actual production, the most important performance requirements to determine the appropriate process parameters can be obtained based on the application of products.

(2) Different process parameters have different degrees of effect on performance. By adjusting the main process parameters, a certain property of the composite material can be greatly changed.

(3) The same process parameters have different degrees of effect on different properties, the important properties should be addressed first, before determining the process conditions.

(4) For silica/carbon black filled rubber, the tensile strength is not only affected by the dispersion of the filler, but also depends on the chemical bond content between the filler and the rubber. Additionally, the initial mixing module affects the dispersion and distribution of the filler, as well as the mixing degree of downstream reaction. Notably, silica and silane coupling agents cannot react without contact. For reaction mixing, the appropriate mechanical dispersion force is significant, as well as the reaction time and reaction temperature.

(5) Based on the consideration of the four properties of filler–filler interaction, tensile strength, chemical reinforcing effect and tear strength, the optimal serial modular continuous mixing process parameters of reinforcing natural rubber composite materials with silica/carbon black are determined as: initial mixing rotor speed 45 rpm, initial mixing temperature 50 °C, filling factor 0.77, initial mixing time 95 s, reactive mixing rotor speed 40 rpm, feeding barrel temperature 147 °C, reactive barrel temperature 140 °C, extruding barrel temperature 128 °C.

## Figures and Tables

**Table 1:** Rubber formula and materials.

Supplier	Material	Content (phr ^1^)
Von Bundit Co., Ltd., Phuket, Thailand	Natural rubber STR20	100
CABOT, Boston, MA, USA)	Carbon black N330	38.5
SOLVAY, Brussels, Belgium	Silica	15
Nanjing Shuguang Chemical Group Co., Ltd., Nanjing, China	TESPT ^2^	2
SINOPEC, Beijing, China	Zinc Oxide	3.5
	Stearic Acid	2
Rhein Chemie Rheinau GmbH, Mannheim, Germany	Microcrystalline Wax	1
China Sunsine Chemical Holdings Ltd., Heze, China	DMPPD ^3^	2
	TMQ ^4^	1.5
	Plasticizer A	2
	TBBS ^5^	1.25
	Sulfur	1

^1^ phr (parts per hundreds of rubber) is the number of additives per 100 (by mass) of rubber. ^2^ TESPT is the abbreviation of Bis-[3-(triethoxysilyl)propyl]tetrasulfide. ^3^ DMPPD is the abbreviation of N-(1,3-Dimethylbutyl)-N-phenylparaphenylenediumine. ^4^ TMQ is the abbreviation of 2,4-diamino-5-methyl-6-[(3,4,5-trimethoxyanilino)methyl] quinazoline. ^5^ TBBS is the abbreviation of N-t-butyl-2-benzothiazole sulfonamide.

**Table 2:** Rubber formula and materials.

Factors		A	B	C	D	E	F	G	H	I
Levels		Initial Mixing Rotor Speed (rpm)	Initial Mixing Temperature (°C)	Filling Factor	Initial Mixing Time (s)	Reactive Mixing Rotor Speed (rpm)	Feeding Barrel Temperature (°C)	Reactive Barrel Temperature (°C)	Extruding Barrel Temperature (°C)	Error Term
1		30	30	0.6	70	20	140	130	120	(1) ^1^
2		40	40	0.7	90	30	145	135	125	(2)
3		50	50	0.8	110	40	150	140	130	(3)
4		60	60	0.9	130	50	155	145	135	(4)

^1^ There is no specific value of levels for the error term. To distinguish the four levels, it is expressed as (1)–(4).

**Table 3:** Orthogonal test schedule.

Factors		A	B	C	D	E	F	G	H	I
Number		Initial Mixing Rotor Speed (rpm)	Initial Mixing Temperature (°C)	Filling Factor Initial	mixing Time (s)	Reactive Mixing Rotor Speed (rpm)	Feeding Barrel Temperature (°C)	Reactive Barrel Temperature (°C)	Extruding Barrel Temperature (°C)	Error Term
1		30	30	0.6	70	20	140	130	120	(1) ^1^
2		30	40	0.7	90	30	145	135	125	(2)
3		30	50	0.8	110	40	150	140	130	(3)
4		30	60	0.9	130	50	155	145	135	(4)
5		40	30	0.6	90	40	150	145	135	(4)
6		40	40	0.7	70	50	155	140	130	(3)
7		40	50	0.8	130	20	140	135	125	(2)
8		40	60	0.9	110	30	145	130	120	(1)
9		50	30	0.7	110	20	145	140	135	(4)
10		50	40	0.6	130	30	140	145	130	(3)
11		50	50	0.9	70	40	155	130	125	(2)
12		50	60	0.8	90	50	150	135	120	(1)
13		60	30	0.7	130	40	155	135	120	(1)
14		60	40	0.6	110	50	150	130	125	(2)
15		60	50	0.9	90	20	145	145	130	(3)
16		60	60	0.8	70	30	140	140	135	(4)
17		30	30	0.9	70	30	150	135	130	(3)
18		30	40	0.8	90	20	155	130	135	(4)
19		30	50	0.7	110	50	140	145	120	(1)
20		30	60	0.6	130	40	145	140	125	(2)
21		40	30	0.9	90	50	140	140	125	(2)
22		40	40	0.8	70	40	145	145	120	(1)
23		40	50	0.7	130	30	150	130	135	(4)
24		40	60	0.6	110	20	155	135	130	(3)
25		50	30	0.8	110	30	155	145	125	(2)
26		50	40	0.9	130	20	150	140	120	(1)
27		50	50	0.6	70	50	145	135	135	(4)
28		50	60	0.7	90	40	140	130	130	(3)
29		60	30	0.8	130	50	145	130	130	(3)
30		60	40	0.9	110	40	140	135	135	(4)
31		60	50	0.6	90	30	155	140	120	(1)
32		60	60	0.7	70	20	150	145	125	(2)

^1^ There is no specific value of levels for the error term. To distinguish the four levels, it is expressed as (1)–(4).

**Table 4:** Range analysis of orthogonal experiments based on the Payne effect test results.

Factors	A	B	C	D	E	F	G	H	I
	Initial Mixing Rotor Speed (rpm)	Initial Mixing Temperature (°C)	Filling Factor	Initial Mixing Time (s)	Reactive Mixing Rotor Speed (rpm)	Feeding Barrel Temperature (°C)	Reactive Barrel Temperature (°C)	Extruding Barrel Temperature (°C)	Error Term
$\bar{K_{1}}$ ^1^	344.8	334.7	374.6	336.9	369.0	333.1	326.8	348.9	321.6
$\bar{K_{2}}$	338.2	341.2	298.8	325.5	319.4	345.2	361.5	297.5	330.5
$\bar{K_{3}}$	332.8	339.4	315.9	336.5	348.3	322.7	332.0	326.1	343.1
$\bar{K_{4}}$	356.2	356.7	382.7	373.0	335.2	371.0	315.6	399.4	326.8
Range	23.4	22.0	83.8	47.5	49.6	48.3	34.7	101.9	21.6
Optimal levels	H2 ^2^ C2E2 F3 D2 G4 A3 B1 (in descending order of major and minor)								

^1^$\bar{K_{1}}$-$\bar{K_{4}}$ represents the average of indicators at each level of each factor. ^2^ The letters A–H represent 8 factors, and the numbers 1-4 represent 4 levels.

**Table 5:** Range analysis of orthogonal experiments based on the tensile strength test results.

Factors	A	B	C	D	E	F	G	H	I
	Initial Mixing Rotor Speed (rpm)	Initial Mixing Temperature (°C)	Filling Factor	Initial Mixing Time (s)	Reactive Mixing Rotor Speed (rpm)	Feeding Barrel Temperature (°C)	Reactive Barrel Temperature (°C)	Extruding Barrel Temperature (°C)	Error Term
$\bar{K_{1}}$ ^1^	28.494	28.754	28.308	28.421	28.51	28.366	28.599	28.821	28.486
$\bar{K_{2}}$	28.786	28.534	28.654	28.68	28.545	28.971	28.495	28.442	28.744
$\bar{K_{3}}$	28.425	28.444	29.083	28.371	29.02	28.523	28.345	28.475	28.345
$\bar{K_{4}}$	28.381	28.355	28.042	28.614	28.011	28.226	28.648	28.348	28.549
Range	0.405	0.399	1.041	0.309	1.009	0.745	0.303	0.473	0.399
Optimal levels	C3 ^2^ E3 F2 H1 A2 B1 D2 G4 (in descending order of major and minor)								

^1^$\bar{K_{1}}$-$\bar{K_{4}}$ represents the average of indicators at each level of each factor. ^2^ The letters A–H represent eight factors, and the numbers 1–4 represent four levels.

**Table 6:** Range analysis of orthogonal experiments based on the M300/M100test results.

Factors	A	B	C	D	E	F	G	H	I
	Initial Mixing Rotor Speed (rpm)	Initial Mixing Temperature (°C)	Filling Factor	Initial Mixing Time (s)	Reactive Mixing Rotor Speed (rpm)	Feeding Barrel TemperaTure (°C)	Reactive Barrel TemperaTure (°C)	Extruding Barrel Temperature (°C)	Error Term
$\bar{K_{1}}$. ^1^	5.549	5.679	5.895	5.693	5.587	5.708	5.932	5.876	5.806
$\bar{K_{2}}$	6.047	5.909	5.913	5.897	5.906	5.823	5.992	5.836	5.834
$\bar{K_{3}}$	6.107	5.943	5.69	5.937	5.89	6.008	6.006	5.903	5.849
$\bar{K_{4}}$	5.595	5.938	5.8	5.771	5.915	5.759	5.669	5.683	5.809
Range	0.558	0.264	0.223	0.244	0.328	0.3	0.337	0.22	0.043
Optimal levels	A3 ^2^ G3 E4 F3 B3 D3 C2 H3 (in descending order of major and minor)								

^1^$\bar{K_{1}}$-$\bar{K_{4}}$ represents the average of indicators at each level of each factor. ^2^ The letters A-H represent eight factors, and the numbers 1-4 represent four levels.

**Table 7:** Range analysis of orthogonal experiments based on the tear strength test results.

Factors	A	B	C	D	E	F	G	H	I
	Initial Mixing Rotor Speed (rpm)	Initial Mixing Temperature (°C)	Filling Factor	Initial Mixing Time (s)	Reactive Mixing Rotor Speed (rpm)	Feeding Barrel Temperature (°C)	Reactive Barrel Temperature (°C)	Extruding Barrel Temperature (°C)	Error Term
$\bar{K_{1}}$ ^1^	137.63	130.88	125.63	132.63	136.88	130.88	133.88	129	134.25
$\bar{K_{2}}$	134.63	134.13	141.13	135.25	138.25	136.38	132.88	137.88	134.54
$\bar{K_{3}}$	135.00	142.13	141.63	140.25	134.38	136.00	140.88	139.25	136.96
$\bar{K_{4}}$	134.63	134.75	133.50	133.75	132.38	128.63	134.25	135.75	135.92
Range	3.00	11.25	16.00	7.63	5.88	7.75	8.00	10.25	2.71
Optimal levels	C3 ^2^ B3 H3 G3 F2 D3 E2 A1 (in descending order of major and minor)								

^1^$\bar{K_{1}}$-$\bar{K_{4}}$ represents the average of indicators at each level of each factor. ^2^ The letters A-H represent eight factors, and the numbers 1–4 represent four levels.

**Table 8:** Optimal process parameters corresponding to each property.

Factors	Initial Mixing Rotor Speed (rpm)	Initial Mixing Temperature (°C)	Filling Factor	Initial Mixing Time (s)	Reactive Mixing Rotor Speed (rpm)	Feeding Barrel Temperature (°C)	Reactive Barrel Temperature (°C)	Extruding Barrel Temperature (°C)
Payne effect	48⑦ ^1^	30⑧	0.77②	95⑤	30③	150④	145⑥	126①
Tensile strength	40⑤	30⑥	0.78①	90⑦	40②	145③	145⑧	120④
M300/M100	45①	40/50/60⑤	0.68⑦	100⑥	30/4/50③	147④	140②	128⑧
Tear strength	30⑧	50②	0.75①	110⑥	30⑦	147⑤	140④	128③

^1^ The major and minor rankings of the effect of the process parameters on the performance are represented by ①–⑧ in the table, where ① is the most major, and ⑧ is the most minor.
